# The Influence of Active Compounds of Chips Made from Different Wood Species on the Antioxidant, Oenological and Sensory Properties of Apple Wines

**DOI:** 10.3390/molecules29132972

**Published:** 2024-06-22

**Authors:** Tomasz Tarko, Aneta Pater, Magdalena Januszek, Aleksandra Duda, Filip Krankowski

**Affiliations:** Department of Fermentation Technology and Microbiology, Faculty of Food Technology, University of Agriculture in Krakow, ul. Balicka 122, 30-149 Krakow, Poland; aneta.pater@urk.edu.pl (A.P.); magdalena.januszek@urk.edu.pl (M.J.); aleksandra.duda@urk.edu.pl (A.D.); filip.krankowski@student.urk.edu.pl (F.K.)

**Keywords:** active wood compounds, wood chips, apple wines, wine ageing (maturation), antioxidant activity, polyphenolic compounds, volatile compounds

## Abstract

Wood chips contain numerous active compounds that can affect the wine’s characteristics. They are commonly used in red grape wines, whisky, cherry and brandy, but in fruit wines, production is not typically utilised. The aim of this study was to compare the impact of an oak barrel ageing with the effect of the addition of chips made from various types of wood (oak, maple, cherry, apple) and with various degrees of toasting to the apple wines on their antioxidant, oenological and sensory properties. The oenological parameters, the polyphenols content, antioxidant activity and content of volatile odour-active compounds were assessed. It was shown that ageing in the presence of wood chips had a less noticeable effect on the oenological and sensory parameters of the wine than barrel ageing. Moreover, wood chips used did not significantly affect the acidity, alcohol and extract content of apple wines. Wines aged in the presence of oak chips (particularly lightly toasted) exhibited the greatest increase in polyphenols, while the polyphenol content of wines aged in the presence of other chips was not dependent on their toasting degree. The ageing of fruit wines with wood chips influences the volatile profile and the olfactory sensations, which can improve their quality.

## 1. Introduction

In ancient times, the contact of food products with wood was primarily related to their storage and transport. Drinks and other food products were stored and transported in wooden barrels, which were relatively light and durable [1,2]. Currently, wood is used primarily because of its interesting and valuable active compounds that shape the aroma, taste and colour of drinks. Currently, acid-resistant steel tanks and containers are employed for both the ageing and transport of wine. Barrels are primarily used for the ageing of alcoholic beverages, particularly those with distinctive qualitative and sensory characteristics, including grape wines, whiskey, brandy and cognac. The chemical compounds derived from wood exert a profound influence on the properties of these beverages, including their antioxidant activity, taste and smell [3,4].

The active ingredients derived from wood can be divided into two categories: those occurring naturally in the wood and those resulting from thermal treatment. Oak wood, which is most often used for wine maturation, contains approximately 45% cellulose, 25% hemicellulose, 20% lignin and 10% of other active compounds that are of particular importance in winemaking. These include alcohols, esters, terpenes, steroids, terpenoids, lactones and phenols [5,6]. Phenolic compounds, particularly those of low molecular weight, impart astringency and bitterness to beverages and turn them darker in colour.

Furthermore, they influence the antioxidant potential of beverages. 4-Ethylphenol and 4-vinylphenol smell similar to medicines and leather, respectively [7]. Guaiacol and its derivatives (4-methylguaiacol, 4-ethylguaicol and 4-vinylguaiacol) impart an aroma described as smoky, spicy, toasted, oak and clove-like [6,8]. Among the phenolic compounds, ellagitannins are most easily extracted by wine and other alcoholic beverages. Castalagin, vescalagin, grandinin and roburin have strong antioxidant properties but also impart astringency to wines [9,10,11,12]. Lactones, which occur in very low concentrations, also contribute to the sensory profile of wines, imparting notes of coconut and wood due to their low sensory detection threshold (20–370 µg/L) [2,5]. The toasting of wood results in the partial thermodegradation of wood components, which alters the profile of active compounds extracted during wine maturation. Modifying the temperature (from 120 to 230 °C) and thermal treatment times (from 15 to 90 min) resulted in varying degrees of alteration in the properties of wood, which consequently led to a variable profile of active compounds in the chips [1,13]. The most susceptible to the temperature impact are lignins, hemicelluloses and ellagitannins [14]. As a result of their decomposition, phenolic aldehydes and ketones, volatile phenols, acids and lactones are formed, giving wines the aroma of smoke, wood and vanilla. Thermally treated polysaccharides are precursors of furan compounds responsible for the aroma of bread, nuts and caramel [1,15]. Furthermore, the effect of temperature has been observed to reduce the concentration of terpenoids, which may contribute to the lower antioxidant potential of extracts [16].

Wooden barrels are expensive and difficult to obtain, and their use results in the loss of drink due to evaporation through the porous structure of the wood. An alternative is to use wood chips. This solution is much cheaper, and the contact surface of the wine with the wood chips is more developed than when using barrels [3,17]. The use of wood chips also allows the use of other than oak tree species when ageing wines and other alcoholic beverages. Other wood species are typically distinguished by their greater porosity, which consequently affects the extraction of active compounds from wood into the beverage. The composition of wood species other than oak also varies. Wood obtained from cherry (*Prunus avium*) and false acacia (*Robinia pseudoacacia*) is distinguished by a higher content of condensed tannins, flavonoids and an altered phenolic acid profile. Chestnut wood (*Castanea sativa*) is characterised by high hardness, similar to oak, but with a larger amount of active compounds that exert a clear influence on the aroma, taste and colour of drinks aged with it [18,19,20,21,22].

Fruit wines have the potential to arouse consumer interest, although they are still considered to be of a lower quality than grape wines. However, many fruit wines are a rich source of aroma compounds, as well as antioxidant ingredients and polyphenols. Fruit wines are obtained in a similar way to grape wines, although additional technological procedures must be used in order to obtain wines of comparable quality to grape wines [23]. The most prevalent type of fruit wine is apple wine. Apples are cultivated on most continents and are a suitable raw material for wine production. The compounds responsible for the aroma of apple wines, including esters, higher alcohols, fatty acids, aldehydes, ketones, terpenes and lactones, are created during fermentation with the participation of yeast and bacteria. However, their profile is also closely related to the type and concentration of aromatic compounds originating from the fruit. Furthermore, their profile is also dependent on the time and conditions of ageing [24,25,26,27].

The scientific literature clearly shows that fruit wines are rarely aged in contact with wood, especially apple wines. In our previous study [28], we demonstrated, among other things, that the ageing of apple wines in the presence of chips made from oak significantly increased the antioxidant activity and polyphenol content in these beverages. Furthermore, we found that these changes depended on the level of chip toasting. Moreover, although the addition of wood chips had no significant impact on the ethanol content, wine extract and colour, it caused a significant change in volatile acidity and the scores of sensory assessment. Wine is typically aged in barrels for a minimum of one year. However, the use of wood chips during the maturation enables a considerable acceleration of this process, due to the increased contact surface area between the chips and wine. The research hypothesis underlying the study was that shorter ageing of apple wines through the use of wood chips, regardless of the species of wood from which they are made, is an attractive alternative to ageing wines in oak barrels. Consequently, a comparison was conducted between the ageing of apple wines in the presence of wood chips and that in barrels. The specific objective of the present study was to compare the effect of 1-month ageing of apple wines in the presence of wood chips made from various tree species (oak, maple, cherry, apple) and of various degrees of toasting (lightly-, medium- and heavily toasted) with the 1-year lasting ageing in oak barrel on the antioxidant, oenological and sensory properties of wines. 

## 2. Results

### 2.1. Oenological Parameters

In order to assess the oenological parameters of apple wines matured in the presence of chips of various tree species and in oak barrels, the total acidity, alcohol content and extract content were analysed. The results are presented in Table 1. 

The total acidity of wines aged in the presence of wood chips ranged from 7.64 to 7.71 g of malic acid per litre, with all cases exhibiting significantly lower values than those observed in control apple wine aged for one year in an oak barrel (7.75 g of malic acid/L). It should be highlighted that the results were consistent and the acidity of the wines was not dependent on the type of wood used. In the case of additions of heavily toasted oak wood chips, a lower acidity value was observed than in the case of lightly- and medium-toasted oak chips. Conversely, heavily toasted apple wood chips increased the acidity of apple wines. However, both of these differences were not statistically significant. 

Two key parameters of wines are alcohol content and extract content. The alcohol content of all wines aged in contact with wood chips was found to be consistently homogeneous, with values ranging from 11.52 to 11.57% (Table 1). It was demonstrated that neither the type of wood from which the chips were made nor the level of toasting had any effect on the alcohol concentration in apple wines. It is of significant importance to note that the alcohol content of wines that had been aged in oak barrels for one year was found to be statistically significantly lower, reaching 11.21%.

The content of total extract in apple wines matured in the presence of chips of various tree species was found to be equal, with no statistically significant differences (Table 1). Similarly to the alcohol content, there was no significant influence of the degree of toasting of the wood chips on the level of wine extract. However, the ageing of the wines in oak barrels contributed to a significant (over 5%) increase in the extract content.

### 2.2. Polyphenol Content and Antioxidant Activity of Apple Wines

The content of total polyphenols and antioxidant activity was determined in wines aged in the presence of wood chips and in oak barrels (Table 2).

The results demonstrated a notable degree of variability. The ageing of apple wines in the presence of oak wood chips was found to result in the greatest increase in polyphenol content (Table 2), with the greatest variation observed in the case of lightly toasted chips. The analysis revealed that wines aged with oak chips exhibited a higher concentration of polyphenols than those aged with maple, apple and cherry chips, with the difference reaching 40%, 41% and 54%, respectively. Notably, wines aged with lightly toasted cherry chips exhibited the lowest total polyphenol content. In the case of oak and maple chips, a decrease in the polyphenol content was observed with an increasing degree of toasting, although the differences were statistically significant only for wines with oak chips. The polyphenol content of wines aged in the presence of apple and cherry chips was not found to be dependent on the degree of chips toasting. It was observed that the ageing of wines in oak barrels significantly (over fourfold) increased the content of total polyphenols, in comparison to wines aged in the presence of oak chips.

The highest antioxidant activity was observed in wines aged with oak wood chips (Table 2). In the case of other wood species, the antioxidant potential of the wines was more uniform, and the observed differences were not statistically significant. Wines aged in the presence of heavy-toasted wood chips exhibited lower antioxidant activity than wines aged with medium-toasted wood chips, although the observed differences were statistically significant only in the case of oak chips. Apple wines aged in oak barrels exhibited an antiradical potential that was over four times higher than wines aged in the presence of oak chips (Table 2). The antioxidant activity of the wines was found to be closely correlated with the total polyphenol content, with a correlation coefficient of 0.99.

### 2.3. Colour Parameter (CIELab) of Apple Wines

The L* value indicates the brightness of the sample (the concentration of pigments) and can take values from 0 (pure black) to 100 (pure white). All wines aged with wood chips were characterised by high brightness (L* above 90), and the observed differences were not statistically significant. Only wines matured in barrels showed values lower than 90 (Table 3), and these values were statistically lower than the samples with wood chips.

Significantly more variation in results was observed for the a* and b* values. With regard to green and red colours (a*), no statistically significant differences were observed within the wood chips produced from the same wood species. However, wines with apple wood chips exhibited a slight shift in colour towards green. Wines aged in barrels exhibited a slight shift in the parameter a* towards the red colour, in comparison to wines with wood chips (Table 3). The parameter b* was found to be significantly shifted towards yellow in wines aged in barrels. Furthermore, wines matured with lightly toasted oak chips exhibited a more intense yellow colour than those matured with more strongly toasted chips. All other samples, matured with wood chips of other wood types, were characterised by a similar value of the b* parameter.

### 2.4. The Content of Volatile Compounds in Apple Wines

The profile of volatile compounds responsible for the odour was determined using the SPME-GC-MS method (Table 4). Additionally, odour activity values (OAVs) were calculated by dividing the concentrations of aroma compounds by their sensory thresholds (taken from the literature). Only volatiles with an OAV > 1 can be perceived and contribute to the overall aroma of the wine. Therefore, the concentration values that give an OAV greater than 1 are highlighted in green in the table.

The wines were dominated by alcohols, primarily amyl alcohols, isobutyl alcohol and phenylethyl alcohol. Esters, terpenes and aldehydes occurred in lower concentrations (Table 4). 

The highest concentrations of the tested alcohols were detected in wines aged with apple wood chips, but wines with oak chips also contained significant amounts of alcohol that contributed to their aroma. In most wines with apple and oak wood chips, the concentrations of the tested alcohols were highest after using moderately toasted wood chips (Table 4). The concentration of undecanol increased with the degree of burning of wood chips from most tree species, except maple. In this case, its concentration decreased as the degree of burnout increased.

Wines aged with chips made from fruit trees (cherry, apple) were found to have relatively higher concentrations of most esters. Oak and maple wood, on the other hand, contained smaller amounts of volatile compounds, imparting the wines a fruity aroma. The concentration of most of the analysed esters was found to increase as the degree of toasting of the chips increased, and in some cases, stronger toasting was necessary for the appearance of some esters (Table 4).

The concentration of the tested terpenes in apple wines was more uniform and less dependent on the type of wood, particularly in the case of α-pinene and limonene. However, wines aged with apple wood chips exhibited higher levels of esters compared to other types of wood chips. Additionally, cherry wood contributed the smallest amounts of terpenes to apple wines (Table 4). The higher degree of toasting of the chips from the wood species used contributed to the presence of higher amounts of terpenes in apple wines.

The concentration of furfural, responsible for the roasted aroma, was found to be highest in wines exposed to medium-toasted wood chips. The concentration of benzene acetaldehyde in the wines did not appear to be influenced by the type of wood used or the degree of toasting, and the observed differences were not statistically significant. However, the amount of nonanal increased with the degree of burning of the chips, with the exception of maple wood (Table 4).

The concentration of volatile compounds that contribute to the aroma of barrel-aged apple wines was relatively low, despite the much longer duration of this process compared to ageing with wood chips (Table 4).

Within the group of identified compounds, concentrations above the threshold (OAV > 1) were found for isoamyl alcohol, ethyl and propyl butanoate, hexyl acetate, ethyl hexanoate and nonanal, whereas in the case of wines matured with apple and oak wood chips, also for some terpenes.

### 2.5. Analysis of Volatile Compounds Affecting the Aroma of Apple Wines

The olfactometric analysis demonstrated a direct correlation between the aromatic activity of a given compound and its concentration in the tested samples (Table 5). The higher the concentration, the greater the aromatic strength of the compound. Some compounds were found to be aromatically active in all samples, including, among others, 3-methyl-1-butanol and ethyl hexanoate (their obtained concentrations were above the sensory threshold, which is confirmed by odour activity values (OAVs), isobutyl alcohol, phenylethanol, ethyl acetate, ethyl propanoate, methyl butanoate, furfural and benzene acetaldehyde.

The highest activity was observed in all samples for ethyl acetate, which exhibited a pineapple aroma. The remaining compounds demonstrated aromatic activity when specific types of wood chips were employed. An illustrative example is undecanol, the scent of which was discerned in samples aged with cherry chips, and the intensity of the aroma was found to be consistent regardless of the degree of toasting. Another compound was ethyl butanoate, whose aromatic activity was observed in wines aged with the addition of cherry and apple chips (irrespective of the degree of roasting) and in wines aged in barrels.

In certain instances, the degree of burning of the chips was a primary factor that exerted a considerable influence on the aromatic activity of a given compound. An illustrative example is geraniol, the scent of which was identified only in two wines that had been aged with the addition of cherry and oak chips with the highest degree of toasting. Among all the wines that were analysed, the highest aromatic activity was demonstrated by wines that had been aged with the addition of heavily toasted cherry chips.

**Figure 1:**
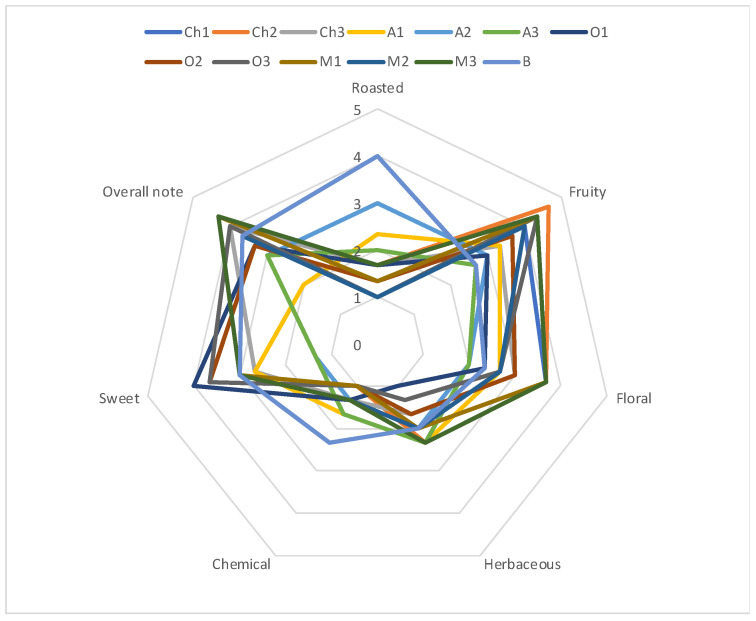
Sensory analysis (QDA) of the apple wines aged in barrel (B) or with the addition of various wood chips (Ch—cherry wood chips, A—apple wood chips, O—oak wood chips, M—maple wood chips; 1—lightly toasted, 2—medium-toasted, 3—heavily toasted); n = 5; STD <5%.

All the wines obtained were subjected to sensory analysis (QDA) (Figure 1). The highest overall rating (almost 4.5/5 points) was awarded to wines aged with the heavily toasted maple wood chips, which also received high scores in the floral and fruity categories. Wines aged with medium-toasted cherry wood chips received the highest score for fruit aroma. The occurrence of a fruity aroma could be related mainly to the aromatic activity of ethyl acetate but also to 3-methyl 1-butanol, ethyl propanoate, methyl butanoate and ethyl hexanoate. Floral aromas were associated with the presence of phenylethanol and geraniol, citronellol and nonanal. In turn, wines aged with lightly toasted oak wood chips were characterised by high sweetness. Wines aged in barrels received the highest ratings in the roasted and chemical categories. It could be related to the aromatic activity of isobutanol and furfural. The other wines analysed received high scores in the fruity, floral and sweet categories.

## 3. Discussion

### 3.1. Oenological Parameters of Apple Wines

During fermentation, yeasts metabolise sugars present in the apple must, synthesising mainly ethanol, carbon dioxide and other by-products that determine the quality of wines [30]. The quantity of ethanol in wine is primarily influenced by the initial amount of sugars present in the setting, originating from the fruit and added during the technological process. The alcohol content is also influenced by the yeast strain employed, the temperature at which the fermentation process occurs and the quantity of sulphur dioxide utilised [31]. The alcohol content of the analysed apple wines, aged with wood chips of varying tree species, with differing degrees of toasting, ranged from 11.52 to 11.57% (Table 1). The results obtained were not statistically different (*p* > 0.05). Similar results were obtained for other works on similar topics [32,33,34,35,36,37]. The lack of change in alcohol concentration after ageing wines with the addition of wood chips is positive and expected. It is reasonable to assume that a small addition of wood chips and wine ageing in tightly sealed vessels will not contribute to a loss of wine strength. On the other hand, the decrease in alcohol concentration in barrel-aged wines is completely natural. The extended maturation period of wines and the porous nature of oak wood contribute to the occurrence of micro-losses of alcohol, which is commonly referred to as the “angel’s share” [38,39].

In dry wines, the optimal taste profile, free from the perceived shortcomings of a “hollow taste”, is largely influenced by the sugar-free extract. This extract is comprised of glycerol, which contributes to the velvety aftertaste observed in fermented beverages due to its oily consistency. Additionally, glycerol plays a role in the viscosity of wines, which can be observed in the glass as the liquid flows down the walls, leaving characteristic “tears” that form a “crown” [40]. The extract content of wines aged in the presence of wood chips from different wood species ranged from 2.95 to 3.00 g/L, with no statistically significant differences observed. The degree of toasting of wood chips also showed no effect on extract content (Table 1). In the study by Călugăr et al. [35], the authors demonstrated that, regardless of the degree of toasting of the wood chips, maturation in oak barrels or maturation in tanks without the addition of wood, the wines exhibited no significant increase or decrease in the total extract. Nunes et al. [37] and Casassa et al. [41] have reported that the addition of oak chips to wines and barrel ageing do not significantly affect total extract content. The present study demonstrated an increase in total extract content in barrel-aged wines (Table 1). This may be attributed to the extraction of compounds, primarily polyphenolic compounds, from the surface of the barrels and the loss of alcohol observed with barrel ageing. The evaporation of some volatile compounds results in a thickening of the extract, thereby increasing its concentration.

The total acidity of wines is primarily derived from tartaric, malic, lactic and citric acids. They originate mainly from fruits but are also formed during the fermentation of wines [42]. According to the legal regulations in Poland [43], the total acidity in fermented fruit beverages should range from 3.5 g to 9 g of malic acid per litre. All apple wines matured in the presence of wood chips met this requirement. No statistically significant differences were found in the total acidity of wines matured with wood chips from different wood species and with different degrees of burnout (Table 1). Martínez-Gil et al. [34] demonstrated that wines treated with French oak chips exhibited a lower titratable acidity compared to wines with the addition of Romanian and American oak. Conversely, Călugăr et al. [35] investigated the impact of natural and lightly toasted oak chips on white grape wines and found that the degree of toasting of oak chips did not significantly affect the overall acidity of the wines. Other studies [17,37,41] have demonstrated that the dosage and type of wood chips do not significantly affect the total acidity of wines. The acidity of wine aged in barrels increased slightly in comparison to wines aged with wood chips. This may be attributed to weight loss through evaporation through the pores of the oak wood and extraction of acids that originate from the oak wood. Furthermore, Călugăr et al. [35] demonstrated that the ageing time of wines affects their total acidity.

### 3.2. The Polyphenol Content and Antioxidant Activity of Apple Wines

Wines contain a variety of polyphenolic compounds, including phenolic acids, flavonols, flavanols, tannins and anthocyanins. The content of polyphenolic compounds in wines is influenced by the species and variety of fruit, the degree of ripeness, fermentation and maturation conditions [44,45]. The ageing of wines results in a change in the qualitative and quantitative composition of polyphenols, which is influenced by a number of factors, including the occurrence of copigmentation, condensation and polymerisation of these compounds [32,46]. The presence of wood (barrels, staves, chips) during the ageing process also affects the profile of polyphenolic compounds in wines. The content of phenolic compounds is contingent upon the species of wood employed, the degree of toasting, the alcohol content of the wines and the contact surface of the beverage with the wood [47,48].

The content of total polyphenols in apple wines aged with wood chips was dependent on the wood species (Table 2). Significantly higher values were found in wines aged with oak chips. Rodríguez Madrera et al. [49] demonstrated a sixfold higher concentration of phenolic compounds in samples extracted from oak wood in relation to cherry wood. However, these were model samples using a 55% hydroalcoholic mixture. The oak wood extracts exhibited a prevalence of syringaldehyde, coniferaldehyde and sinapaldehyde, with concentrations that were 5 to 8 times higher than those observed in the cherry wood extracts. Oak wood contributed gallic acid to the extract, which was absent in cherry wood, but protocatechuic acid was only detected in cherry wood extracts. In experiments on model wines [50], it was demonstrated that the ageing of these wines with oak wood chips resulted in concentrations of total phenolic compounds that were more than four times higher than those observed in wines aged with cherry wood and almost nine times higher than those observed in wines aged with apple wood. However, in the same experiments with real grape wine, the differences were no longer so pronounced. In some types of wine, the differences in total polyphenol content were not statistically significant. In most of the trials, oak wood chips contributed approximately 5% higher levels of total polyphenols, while cherry and apple wood chips contributed approximately 2%. Another study [51] demonstrated that wines matured with oak and cherry wood chips had similar levels of total polyphenols. In the present study, the differences were more pronounced, but the experiments were conducted on apple wine, which contains fewer total polyphenols than grape wines. The results gained after the maturation of wines with apple wood chips were higher than those matured with cherry wood, similar to the study by Toulaki et al. [50]. No studies have yet been conducted on the use of maple wood chips for wine maturation, but this wood does not contribute more phenolic compounds to wines than apple wood. The polyphenolic compounds extracted from wood during the ageing of wines are primarily ellagitannins, including castalagin, vescalagin and grandinin, as well as roburin (A, B, C, D and E) [15,52]. Their quantity diminishes with increasing toasting levels [40,43,53], which may explain the outcomes observed in the present study for samples matured with oak chips. Samples aged in barrels exhibited a significantly higher amount of polyphenolic compounds (Table 2), which was attributable to the much longer barrel ageing time and, above all, to the surface area of the wine in contact with the wood. This was many times greater for such small barrels than for wood chips.

The antioxidant activity of wines matured with wood chips from different wood species was found to be closely correlated with the content of polyphenolic compounds, with a correlation coefficient of 0.99. This suggests that the majority of the wine’s free radical-scavenging capacity is derived from phenolic compounds formed during the vinification and subsequent maturation with the use of wood chips. Furthermore, ellagitannins present in wood may have a significant impact on the free radical-scavenging capacity of wines, as they exhibit strong antioxidant activity [12]. Nikolantonaki et al. [54] demonstrated that the ageing of white wine in the presence of oak wood resulted in a fourfold increase in the concentrations of ellagitannins, a twofold increase in the concentrations of phenolic acids and a fourfold increase in the concentrations of flavan-3-ols. Additionally, experiments on whiskey revealed that the antioxidant activity of drinks matured in oak barrels was enhanced, which was attributed to the presence of phenolic acids [55]. The extractability of these compounds from oak chips in this study was the highest, resulting in significantly higher antioxidant activity in relation to chips from maple, cherry and apple wood (Table 2). The antioxidant activity of wines aged with heavily toasted wood chips was lower than in the case of medium-toasted wood chips (Table 2). This effect may be related to the decomposition of some of the active compounds during wood toasting. It has been demonstrated that a high degree of toasting can result in the complete decomposition of roburin D and E, as well as the thermal degradation of ellagitannins, which significantly reduces the antioxidant activity of drinks [9,10,14].

### 3.3. Colour Parameter (CIELab) of Apple Wines

All wines exhibited high brightness (L* around 90 CIELab units), which is attributed to the low anthocyanin content of the apples. The barrel-aged wines were slightly darker, but the reduction in L* values was rather slight. The a* values were close to zero, which was expected for white wines made from apples. The addition of wood chips and, in particular, barrel ageing resulted in an increase in yellow colour (the b* value), which was not dependent on the degree of toasting of the wood chips.

It has been demonstrated [56] that wines darkened after ageing (lower L*), which was due to the higher phenolic content. In the present study, this is very evident in the barrel-aged sample. In a separate study [57], it was demonstrated that the a* and b* parameters are correlated with the anthocyanins of red wines. Furthermore, it was found that ageing in contact with wood significantly alters these parameters. In contrast, no significant differences in the colour parameters were observed for white wines [58,59]. This discrepancy is most likely attributed to the inherent differences between red and white wines, primarily the maceration of grape skins during their production, which results in a higher concentration of anthocyanins [44].

### 3.4. The Content of Volatile Compounds That Contribute to the Aroma of Apple Wines

The sensory profile of wines is shaped by the volatile compounds present in them. These compounds have different sources of origin. Some of the volatile compounds are introduced with the raw material, which is the fruit must. The composition of the must is related to the species and variety of fruits, ripening conditions, sunlight, etc. First of all, these are terpenes, in which only 10% of the must are in volatile form. The majority of these compounds are linked to saccharides by glycosidic bonds [60,61,62]. The dominant amount of volatile compounds that shape the aroma of wines is formed during fermentation and maturation. This group includes higher alcohols, esters, volatile acids and aldehydes [62,63,64]. An important group of compounds are those formed as a result of wine maturation, during which wine components undergo chemical reactions, such as oxidation or esterification, creating unique aromatic substances. Furthermore, the ageing process in contact with wood also affects the volatile compounds of wines. The structure of wood (porosity, permeability) and its chemical composition (polyphenols, tannins and volatile compounds) determine the biochemical processes occurring during wine ageing, which adds complexity to the aroma [65].

Table 4 and Table 5 list the volatile compounds that shape the odour sensations perceived during olfactometric analysis. The majority of alcohols have a fruity or floral scent. Isobutyl alcohol, in addition to the fruity scent, also produces a chemical aroma. Six-carbon alcohols and 2-nonanol have an herbal scent. The aromas of all esters determined in the olfactometric analysis were described as fruity, with the exception of diethyl butyrate, which had an herbal scent. The identified terpenes had a floral and herbal scent, and furfural was responsible for the roasted aroma. Floral and fruity aromas are most desirable in white and fruit wines [65,66]. They impart freshness to wines and are clearly associated with the raw material from which they originate. In the case of red wines, particularly those that have been aged for an extended period, the importance of floral and fruity notes is diminished [64,67].

Previous studies on the impact of wood chips on the sensory profile have primarily focused on red wines [20,50,68]. The experience in ageing white wines with wood chips has been limited to the assessment of polyphenolic compounds and possibly sensory analysis [51,69]. Toulaki et al. [50] demonstrated in their research on red wine that the profile of volatile compounds aged with wood depends on the type of fruit and the type of wood. In certain instances, wines aged with cherry wood chips exhibited a higher concentration of ethyl acetate than those aged with apple wood, while oak wood had the greatest effect on the production of phenylethanol. Other studies [70] have demonstrated that the maturation of red wines in the presence of cherry wood chips results in higher concentrations of most aroma-forming compounds compared to apple wood chips. However, no such changes were observed in this study. Although some volatile compounds were more abundant in wines aged with cherry chips, in some cases, e.g., 3-methyl-1-butanol, ethyl butanoate and α-pinene, the use of apple wood contributed to their higher concentrations in the wines than cherry wood (Table 4). In wines aged with the addition of apple chips, the presence of ethyl butanoate, which is characterised by a pineapple aroma, had a particularly significant impact on the aroma activity (Table 5). The study by Guerrero-Chanivet et al. [18] demonstrated that oak wood extracts exhibited a greater diversity of volatile compounds than cherry wood extracts. However, some compounds were present in higher concentrations in cherry wood extracts, including palmitic acid, cyclopropyl carbinol and pyranone. These findings were based on wood extracts, not wine. The aforementioned compounds were not identified in apple wines in this study (Table 4 and Table 5). Experiments on rose wine aged with oak and cherry wood chips demonstrated minimal variation in the content of nearly all esters, with the exception of methyl vanillate, which exhibited higher concentrations in wines with oak wood. However, wines aged with cherry chips exhibited higher concentrations of the majority of the tested alcohols [19]. The results of this study yielded disparate findings. While wines aged with cherry chips exhibited elevated concentrations of numerous esters, those aged with oak chips demonstrated reduced levels of alcohol that contribute to aroma.

To date, maple wood has not been employed for the maturation of wines. However, the findings of this study indicate that maple wood does not exert a distinctive influence on the volatile compound profile of apple wines. Only ethyl propanoate demonstrated a discernible impact on the olfactory characteristics of wines, particularly in wines aged with medium- and heavily toasted chips (Table 5).

The results of the comparison between the ageing of apple wine in oak barrels and with oak chips are of interest. The alcohol content of the barrel-aged wine was lower than that of the wines aged with chips (Table 1), which corresponded with the higher total acidity (Table 1) and higher extract content (Table 1). Moreover, the concentration of most volatile compounds that contribute to the aroma of barrel-aged apple wines was relatively low, despite the much longer duration of this process compared to ageing with wood chips (Table 4). The largest statistically significant changes were observed for undecanol, hexyl acetate and methyl acetate, with the content of these compounds in the apple wine aged in the barrel being, respectively, over 10 times, approximately 4 times and 3 times lower than in the wine aged in the presence of oak chips, regardless of the degree of roasting. In contrast, the concentration of 2-methyl-ethyl butanoate ester was on average six times higher after barrel ageing than in the presence of wood chips. It was somewhat unexpected that wines aged in oak barrels for one year did not exhibit any more pronounced differences compared to wines aged with wood chips. The content of the other volatile compounds tested did not differ significantly between wines aged in barrels and those aged with wood chips (Table 4). Furthermore, there was no significant impact of the tested compounds on the results of olfactometric tests. The only notable difference was that wines aged in barrels exhibited a darker colour and a higher proportion of polyphenolic compounds (Table 2), which are responsible for the antioxidant activity of wines (Table 2).

Interesting results were obtained during sensory analysis (QDA) (Figure 1). Of the wines analysed, the wine aged with maple wood chips (heavily toasted) and cherry wood chips (medium-toasted) was given the highest overall note. Furthermore, this wine was characterised by a fruity and floral aroma, which was also confirmed by the olfactometric analysis (Table 5). Interestingly, however, there were quite low concentrations of undecanol and methyl octanoate in these highly sensory-rated wines, even though these compounds are perceived as “fruity”. Also, lower concentrations of linanool oxide were more desirable in the sensory well-rated wines. It can also be noted that wines containing high amounts of propyl butanoate, diethyl butanoate and 2-phenylacetaldehyde were highly rated by the sensory panel. In contrast, the presence of furfural (roasted) and α-pinene (herbaceous) in higher concentrations negatively affected the sensory evaluation results, which may be related to their dominant aroma and to a reduction in the perceptibility of fruit flavours. In turn, the wine aged in a barrel was characterised by a chemical and roasted aroma.

It can therefore be concluded that the use of chips from various trees as an alternative to barrel ageing is a promising avenue for further investigation. This option is considerably less expensive than ageing in oak barrels [3,17].

## 4. Materials and Methods

### 4.1. Material

As the research material apples of the Red Boskoop variety, originating from the pomological orchard of the University of Agriculture in Krakow (Poland), were used.

Ethanol fermentation was conducted using dry yeast Aroma White Wine (Starowar, Halinów, Poland), a yeast strain dedicated to white wines. Yeasts were rehydrated in accordance with the protocol provided by the producer prior to inoculation.

Lightly toasted, medium-toasted or heavily toasted oak, maple, apple and cherry chips (provided by Malinowy Nos, Krakow, Poland) were used in the research. As the control, the apple wine aged in oak barrels was used.

Diammonium salt of the 2,2′-azino-bis (3-ethylbenzothiazoline-6-sulfonic) acid (ABTS diammonium salt), (±)-6-hydroxy-2,5,7,8-tetramethylchromane-2-carboxylic acid (Trolox), a phosphate buffer saline (PBS: 0.01 M phosphate buffer, 0.0027 M potassium chloride, 0.137 M sodium chloride; pH 7.4 at a temperature of 25 °C), anethole, ethyl nonanoate and 4-methyl-2-pentanol and internal standard solution were purchased from SIGMA-Aldrich (Taufkirchen, Germany). The chemicals sodium hydroxide (NaOH), sodium chloride (NaCl), sodium carbonate (Na_2_CO_3_), sodium pyrosulfite (Na_2_S_2_O_5_) and potassium persulfate (K_2_S_2_O_8_) were obtained from POCh (Gliwice, Poland).

### 4.2. Fermentation and Ageing of Apple Wines

The apples were washed, crushed (using a laboratory crusher) and mixed with a pectinolytic preparation (Pectinex^®^ YieldMASH, Novozymes, Warszawa, Poland) in the proportion of 80 µL per kg of pulp and left for 24 h at 20 °C. Subsequently, the must was pressed out of the fruit (hydropress Zottel, Žalec, Slovenia). The apple must after pressing had an extract content of 13.1°Blg and an acidity of 7.84 g of malic acid/L. The must was subjected to correction of the extract (up to 20°Blg) and sulphured (50 mg SO_2_/L).

Then, the must was poured into a 25-litre fermentation vat and inoculated with rehydrated yeast in the amount of 0.3 g/L. The fermentation of the apple must was carried out at 20 °C (± 2 °C) for 28 days.

The wine was decanted from the sediment, poured into glass bottles (0.5 L each) and aged with the addition of oak, maple, apple or cherry chips, in doses of 3 g/L. The wine was aged at 20 °C for one month. As a control, a portion of the wine was poured into three 1.5 L oak barrels and aged for one year (at 20 °C).

All tests and analyses were conducted in at least three physical replicates.

### 4.3. Determination of Extract Content, Alcohol, Total Acidity

The total extract content, ethyl alcohol content and acidity were determined in accordance with the methods recommended by the International Organization of Vine and Wine [71]. This involved the total extract and ethanol content being determined using distillation methods, by density determination with an oscillating densimeter (DMA 4500 M, Anton Paar, Warszawa, Poland), and the total acidity being determined by the potentiometric method (expressed as grams of malic acid/L).

### 4.4. Assessment of Antioxidant Activity [72]

The antioxidant activity (AOX) was determined using an ABTS assay. An active ABTS cation radical was generated in the chemical reaction between the 7 mM ABTS diammonium salt and the 2.45 mM potassium persulfate. In order to terminate the reaction and to stabilise the ABTS cation radical, the solution was kept overnight in the dark at ambient temperature. Prior to analysis, the radical solution was diluted with phosphate-buffered saline (PBS) in such a way that allowed for obtaining the final absorbance of A = 0.70 ± 0.02 (ABTS_0.7_) measured at 734 nm (spectrophotometer BECKMAN DU 650, Beckman Instrument, Inc., Fullerton, CA, USA). One hundred microlitres aliquots of the diluted wine or standards (Trolox) were added to 1 mL of ABTS_0.7_, and the absorbance was measured 6 min after mixing. AOX was calculated using a standard curve obtained by measuring the absorbance of synthetic vitamin E (Trolox) solutions (concentration 10–100 mg/L) and expressed in mg of Trolox/100 mL.

### 4.5. Total Polyphenol Content (TPC)

The total polyphenol content (TPC) was determined by the method previously described in detail by Tarko et al. [72], namely the Folin–Ciocalteu method. Briefly, an amount of 45 mL of redistilled water, 0.25 mL of Folin–Ciocalteu reagent (dissolved in water at a ratio of 1:1 *v*/*v*) and 0.5 mL of 7% Na_2_CO_3_ were added to 5 mL of wine that had been adequately diluted with distilled water. The mixture was then left to stand for 30 min in the dark. Subsequently, the absorbance was measured on a spectrophotometer (λ = 760 nm). The obtained results of TPC were expressed as mg of (+)-catechin/100 mL based on the standard plot.

### 4.6. Determination of Chromatic Characteristics According to CIELab

The colour of the wine was evaluated using tristimulus colorimetry, based on transmittance spectra using equations proposed by the International Commission of Illumination (CIE). The wine was clarified by centrifugation (15 min, 5000 rpm, MPW 350R centrifuge) and the absorption spectrum was measured (380–770 nm, CM-5 spectrophotometer, Konica Minolta, Tokyo, Japan). The CIELab parameters were calculated. Coordinate L* represents a perceptual brightness, ranging from 0 (black) to 100 (white), while a* and b* are chromatic coordinates. The a* value is relative to the green/red colour component, with negative values toward green (−100) and positive values toward red (+100), while the b* value represents the blue/yellow colour component, with negative numbers toward blue (−100) and positive toward yellow (+100) [73].

### 4.7. Analysis of the Volatile Compounds Profile of Apple Wines-SPME-GC-MS (Solid-Phase Microextraction–Gas Chromatography–Mass Spectrometry)

About 2 mL of a saturated saline solution mixed with an internal standard solution (4-methyl-2-pentanol at 5 mg/L and ethyl nonanoate at 0.05 mg/L, sourced from Sigma-Aldrich, Saint Louis, MO, USA) and 0.05 mL of the wine sample were combined in a 10 mL vial. Initially, the SPME device from Supelco Inc. (Bellefonte, PA, USA), coated with a PDMS (100 µm) fibre, was preconditioned by heating it in the GC injector port at 250 °C for one hour. The fibre was then exposed to the sample’s headspace, with stirring at 300 rpm for 30 min at 40 °C. Subsequently, the SPME device was inserted into the injector port of an Agilent Technologies 7890B chromatograph system, equipped with a LECO Pegasus HT High-Throughput TOFMS and held in the inlet for 3 min. The GERSTEL MultiPurpose Sampler (MPS, GERSTEL Inc., Linthicum, WA, USA) facilitated the SPME procedure.

Volatile compounds were separated using an Rtx-1ms cross-bond 100% dimethyl polysiloxane capillary column (30 m × 0.53 mm × 0.5 µm), with the detector temperature set at 250 °C. The temperature of the column was initially set at 40 °C for a period of 3 min, after which it was increased at a rate of 8 °C per minute until it reached 230 °C, where it was maintained for a further 9 min. The flow of helium was maintained at 1.0 mL/min. The electron impact mass spectra (EIMS) energy was established at 70 eV, with the ion source and connection parts heated to 250 °C. The analytes were transferred in splitless mode, and the mass storage device (MSD) operated in SIM mode for m/z = 30 to m/z = 300. Volatile compounds were identified through mass spectral libraries and linear retention indices (LRIs), computed from a series of n-alkanes (C6 to C30). A semi-quantitative analysis was executed by measuring the relative peak area of each identified volatile compound using the NIST database, with the internal standard serving as a reference.

The table also includes the thresholds for each compound as specified in the literature [29]. The odour activity value (OAV) is commonly used to determine the “weight” of an odorant’s perception. This value is calculated by dividing the abundance of the odorant molecule by its odour detection threshold. Values greater than 1 mean that the volatile compound can be perceived and are highlighted in green in the table.

### 4.8. The Examination of Volatile Substances by GC-FID-O (Gas Chromatography–Flame Ionisation Detector with Olfactometry)

The identification and analysis of aroma-active volatile compounds in wines were performed using olfactometry techniques with a Hewlett Packard 5890 Series II chromatography system. All samples of wines matured in the presence of woodchips and in barrels were analysed in a minimum of three physical replicates. A mixture containing 2 mL of the wine sample and 1 g of NaCl was placed into a 10 mL vial. The aforementioned mixture was subjected to extraction for 40 min at a temperature of 40 °C. This was achieved by employing a divinylbenzene/carboxen/polydimethylsiloxane (DVB/CAR/PDMS) solid-phase microextraction (SPME) fibre with dimensions of 50/30 µm, provided by Supelco/Sigma-Aldrich (Bellefonte, PA, USA). Subsequently, the SPME apparatus was introduced into the injection port, where it was held at the inlet for a period of 3 min. The separation of analysed compounds occurred on an Rxi^®^-1ms capillary column featuring Crossbond 100% dimethyl polysiloxane, with dimensions of 30 m × 0.53 mm × 0.5 µm. The detector’s temperature was maintained at 250 °C. The analysis programme commenced at 35 °C for a period of 4 min, after which a temperature increase of 5 °C per minute was applied up to 110 °C. This was followed by a further increase of 20 °C per minute to 230 °C, after which the temperature was maintained at this level for a further 4 min. Helium was employed as the carrier gas, with a constant flow rate of 1.0 mL per minute. The process of identifying volatile compounds and internal standards (anethole, ethyl nonanoate and 4-methyl-2-pentanol) was based on the comparison of retention times and peak areas. The concentration of these compounds was adjusted to reflect the 100% (*v*/*v*) ethanol content. An olfactory detection port (ODP-3, Gerstel, Linthicum Heights, Maryland, USA) was employed for olfactometric analysis, through which three expert GC-O analysts assessed and described the aroma-active compounds in terms of type and intensity (ranging from not detected to strong). Three experienced GC-O analysts evaluated the odours they detected and rated their intensity using a 4-point scale (undetected, faint, moderate and intense). The odour-active compounds in each wine sample were identified and classified according to their chemical functional groups. The total odour intensity for each compound class was then calculated.

### 4.9. Sensory Assessments

The sensory analysis of the obtained wines focused on their aromas and included six sensory descriptors: sweet, chemical, herbaceous, floral, fruity and roasted. These were evaluated on a 5-point hedonistic scale in quantitative descriptive analysis (QDA). The panellists were scientific staff from the Faculty of Food Technology and Human Nutrition at the University of Agriculture in Krakow, who had previously graduated from the faculty and completed an extensive course on sensory analysis as part of their curriculum. The panellists were initially presented with standards of different aromas in order to ascertain their ability to recognise each one. Subsequently, they were given the same standards at various concentrations. Only those who successfully completed both stages were selected as panellists. The wines were then subjected to sensory assessments by a panel of 5 panellists. The samples were coded and provided to the panellists in a randomised order.

### 4.10. Statistical Analysis

A minimum of three repetitions of the analysis were conducted, and the results were shown as the arithmetic mean with standard deviation (± SD). The statistical analysis was performed using InStat v. 3.01 (GraphPad Software Inc., San Diego, CA, USA). A one-way analysis of variance (ANOVA) with post hoc Tukey’s test was applied to assess the significance of differences between means. The Kolmogorov–Smirnov test was carried out to assess the normality of the distribution.

## 5. Conclusions

The use of chips of different types of wood, with different degrees of toasting, affects some parameters of apple wines. The oenological parameters of the wines do not change, which can be considered a positive feature. The antioxidant activity of wines was highly correlated with the content of total polyphenols. Wines with oak chips had the greatest impact on the polyphenol content. Other wood chips (maple, apple, cherry) also influenced this parameter but to a lesser extent. The content of polyphenols was found to depend on the degree of toasting of the wood. The concentration of volatile compounds was influenced by the ageing of apple wines in the presence of chips from various wood species, which may shape the final sensory profile of the wines. It is therefore recommended that further research be conducted in order to improve the quality parameters of fruit wines and make them more desirable in the wine market.

## Figures and Tables

**Table 1:** The total acidity, the alcohol content and the extract content of wines that have been aged in the presence of wood.

Parameters	Toasting Level			
	Lightly Toasted	Medium Toasted	Heavily Toasted	Barrel
	1 Month			1 Year
Total acidity [g of malic acid/L]				
Oak	7.66 ± 0.14 ^a^	7.68 ± 0.10 ^a^	7.64 ± 0.00 ^a^	7.75 ± 0.01 ^b^
Maple	7.68 ± 0.04 ^a^	7.68 ± 0.04 ^a^	7.68 ± 0.08 ^a^	—
Apple	7.68 ± 0.10 ^a^	7.68 ± 0.04 ^a^	7.71 ± 0.00 ^a^	—
Cherry	7.68 ± 0.10 ^a^	7.68 ± 0.08 ^a^	7.68 ± 0.04 ^a^	—
Alcohol content [% *v*/*v*]				
Oak	11.52 ± 0.01 ^a^	11.56 ± 0.01 ^a^	11.52 ± 0.01 ^a^	11.21 ± 0.03 ^b^
Maple	11.53 ± 0.01 ^a^	11.54 ± 0.03 ^a^	11.57 ± 0.02 ^a^	—
Apple	11.52 ± 0.09 ^a^	11.56 ± 0.02 ^a^	11.55 ± 0.05 ^a^	—
Cherry	11.56 ± 0.01 ^a^	11.56 ± 0.02 ^a^	11.56 ± 0.02 ^a^	—
Extract content [°Blg]				
Oak	2.98 ± 0.01 ^a^	2.98 ± 0.01 ^a^	2.99 ± 0.01 ^a^	3.15 ± 0.02 ^b^
Maple	2.98 ± 0.00 ^a^	2.99 ± 0.01 ^a^	2.97 ± 0.02 ^a^	—
Apple	2.97 ± 0.00 ^a^	2.97 ± 0.02 ^a^	2.97 ± 0.01 ^a^	—
Cherry	2.95 ± 0.01 ^a^	2.97 ± 0.01 ^a^	2.97 ± 0.03 ^a^	—

The same letters within the analysed parameter indicate no statistical significance for *p* < 0.05.

**Table 2:** The content of polyphenols in wines and their antioxidant activity, which have been aged in the presence of wood.

Parameters	Toasting Level			
	Lightly Toasted	Medium-Toasted	Heavily Toasted	Barrel
	1 Month			1 Year
Total polyphenol content (TPC) [mg of (+)-catechin/100 mL]				
Oak	152.72 ± 4.17 ^a^	142.87 ± 11.48 ^b^	124.08 ± 2.99 ^c^	640.15 ± 24.45 ^g^
Maple	110.89 ± 5.32 ^d,f^	107.48 ± 2.34 ^d,f^	106.73 ± 3.92 ^d,e^	—
Apple	108.24 ± 1.99 ^d,f^	108.94 ± 2.74 ^d,f^	111.88 ± 0.50 ^d^	—
Cherry	98.71 ± 0.10 ^e^	105.72 ± 1.75 ^d,e^	102.65 ± 0.71 ^e,f^	—
Antioxidant activity [mg of Trolox/100 mL]				
Oak	52.77 ± 0.83 ^a^	52.07 ± 0.60 ^a^	45.52 ± 0.41 ^b^	219.87 ± 3.19 ^c^
Maple	42.53 ± 0.98 ^b^	43.64 ± 1.24 ^b^	42.34 ± 0.79 ^b^	—
Apple	42.30 ± 0.17 ^b^	43.02 ± 0.17 ^b^	42.84 ± 1.35 ^b^	—
Cherry	41.73 ± 1.27 ^b^	44.37 ± 0.81 ^b^	43.35 ± 2.96 ^b^	—

The same letters within the analysed parameter indicate no statistical significance for *p* < 0.05.

**Table 3:** The influence of apple wine maturation in the presence of wood on colour parameters (CIELAB).

Wood Species	Toasting Level	L*	a*	b*
Oak	Lightly	94.23 ± 0.41 ^a^	1.28 ± 0.22 ^a^	10.38 ± 1.63 ^a^
	Medium	94.46 ± 0.20 ^a^	0.99 ± 0.20 ^a^	8.53 ± 1.26 ^b^
	Heavily	94.55 ± 0.13 ^a^	0.80 ± 0.15 ^a^	7.59 ± 0.95 ^b^
Maple	Lightly	94.48 ± 0.27 ^a^	0.86 ± 0.30 ^a^	7.39 ± 1.52 ^b^
	Medium	94.46 ± 0.08 ^a^	0.82 ± 0.10 ^a^	7.51 ± 0.77 ^b^
	Heavily	94.51 ± 0.25 ^a^	0.97 ± 0.18 ^a^	8.56 ± 1.07 ^b^
Apple	Lightly	94.72 ± 0.21 ^a^	0.74 ± 0.25 ^b^	7.74 ± 1.79 ^b^
	Medium	94.63 ± 0.25 ^a^	0.79 ± 0.15 ^b^	7.54 ± 0.85 ^b^
	Heavily	94.62 ± 0.10 ^a^	0.73 ± 0.12 ^b^	7.31 ± 0.86 ^b^
Cherry	Lightly	94.36 ± 0.22 ^a^	0.82 ± 0.17 ^a^	7.41 ± 1.09 ^b^
	Medium	94.38 ± 0.10 ^a^	0.95 ± 0.19 ^a^	8.04 ± 1.10 ^b^
	Heavily	94.48 ± 0.19 ^a^	0.83 ± 0.15 ^a^	7.87 ± 0.90 ^b^
Barrel	—	89.27 ± 0.82 ^b^	5.02 ± 0.73 ^c^	42.23 ± 5.52 ^c^

The same letters within the analysed parameter indicate no statistical significance for *p* < 0.05.

**Table 4:** The composition of compounds that contribute to the aroma of apple wines.

Compounds (IUPAC Name)	LRI ^1^	Threshold ^2^	Wood Chips												Oak Barrel	Sig ^3^
			Cherry Lightly Toasted	Cherry Medium-Toasted	Cherry Heavily Toasted	Apple Lightly Toasted	Apple Medium-Toasted	Apple Heavily Toasted	Oak Lightly Toasted	Oak Medium-Toasted	Oak Heavily Toasted	Maple Lightly Toasted	Maple Medium-Toasted	Maple Heavily Toasted		
Alcohols [μg/L]																
Isobutanol (2-methylpropan-1-ol)	617	40,000	48.3 e	2165 c	2300 c	2413 c	5360 a	2724 c	2.24 f	3814 b	2598 c	2.4 f	1034 d	2527 c	2000 c	***
1-butanol (butan-1-ol)	645	150,000	4.0 d	459 b	509 b	535 b	1169 a	571 b	438 b	636 b	553 b	352 c	243 c	556 b	431 b	**
Isoamyl alcohol (3-methylbutan-1-ol)	716	42	10,563 b	10,613 b	11,018 b	8830 c	31,559 a	16,376 b	11,981 b	14,236 b	16,428 b	8888 c	6633 c	13,715 b	10,865 b	**
1-Hexanol (hexano-1-ol)	865	10	nd	2706	2691	3197	3752	2924	2206	2373	2064	nd	1100	2812	2126	ns
5-Hexen-1-ol (hex-5-en-1-ol)	878	nd	nd	nd	nd	7.7	6.3	nd	nd	nd	9.8	4.2	3.1	7.6	nd	ns
2-Nonanol (nonan-2-ol)	1091	nd	nd	nd	10.1	nd	nd	nd	nd	nd	nd	4.1	nd	nd	nd	ns
Phenethyl alcohol (2-phenylethanol)	1102	7020	2032 b	1961 b	2344 b	2877 b	3251 a	2267 b	1933 b	1848 b	1739 b	1828 b	1256 c	2813 b	2038 b	**
Undecanol (undecan-1-ol)	1364	6500	3.7 c	5.7 c	8.9 c	11.3 b	18.3 b	68.9 a	40.1 a	46.3 a	61.9 a	36.3 b	11.4 b	4.3 c	4.2 c	*
Esters [µg/L]																
Ethyl acetate	600	14,000	2546 f	5942 c	6416 c	7696 a	9164 a	7047 b	4214 e	4863 d	4954 d	1243 g	1558 g	6854 c	5034 d	***
Ethyl propanoate	678	1000	1.5 d	143.3 b	150.4 b	158.2 b	148.6 b	90.4 b	92.3 b	334 a	93.7 b	68.4 c	126 b	156 b	108.2 b	**
Methyl butanoate	705	1000	53.2	35.4	32.9	46.1	47.7	46.6	12.7	38.3	28.6	13.1	28.1	29.7	21.9	ns
Ethyl butanoate	789	15	885.2 a	897.1 a	824.1 a	989.9 a	978.7 a	919.3 a	209.2 c	646.3 b	622.5 b	323.5 c	62.6 d	721 b	523.1 b	***
2-methyl-ethyl butanoate (propyl butanoate)	854	15	200.3 a	235.7 a	23.5 c	27.9 c	34.5 c	15.6 c	17.3 c	18.6 c	18.3 c	71.2 b	17.3 c	162.5 a	117.2 a	**
1-Butanol, 2-methyl-acetate (2-methylbutyl acetate)	865	2000	nd	77.2 b	69.8 b	78.8 b	45.4 b	69.5 b	4.5 d	107.2 a	nd	26.4 c	6.4 d	62.5 b	50.3 b	**
Ethyl decanoate	902	8800	nd	141.8	192.5	203.6	171.3	134.1	93.2	59.2	114.9	87.1	158.3	160.4	145.6	ns
Hexyl acetate	996	1.8	19.3 b	21.1 b	18.6 b	20.1 b	19.4 b	20.8 b	39.4 a	43.1 a	44.2 a	9.4 c	8.9 c	11.4 c	9.9 c	*
Ethyl hexanoate	986	14	2652 c	3821 b	3800 b	4611 a	3294 b	3723 b	525.3 d	2121 c	2480 c	13.6 f	82.9 e	2714 c	1843 c	***
Methyl octanoate	1112	1900	14.3 d	64.4 c	116.7 b	219.3 a	193.3 a	158.4 a	148.3 a	154.7 a	174.2 a	34.5 d	3.2 e	93.2 c	56.8 c	***
Diethyl butanoate (diethyl butanedioate)	1149	5000	1365 a	1342 a	1288 a	1412 a	1642 a	1115 a	972.4 b	1159 a	925.4 b	899.9 b	586.6 c	1388 a	967.1 b	*
Terpenes [µg/L]																
α-Pinene (2,6,6-trimethylbicyclo[3.1.1]hept-2-ene)	933	2100	3.7	3.8	6.0	6.8	8.4	7.9	2.5	9.3	3.5	0.6	1.6	3.7	3.8	ns
D-Limonene ((4R)-1-methyl-4-prop-1-en-2-ylcyclohexene)	1020	38	4.3	2.0	2.4	3.3	3.1	1.7	1.5	1.5	1.8	1.9	9.6	2.5	1.9	ns
Linalool oxide (2-[(2R,5S)-5-ethenyl-5-methyloxolan-2-yl]propan-2-ol)	1078	25	4.4 c	3.7 c	3.2 c	38.2 a	48.4 a	40.2 a	18.9 b	44.5 a	41.2 a	20.2 b	6.5 c	16.9 b	13.5 b	**
Camphor (1,7,7-trimethylbicyclo[2.2.1]heptan-2-one)	1126	5000	5.0 c	1.3 c	11.9 c	49.7 a	84.2 a	62.2 a	7.0 c	82.7 a	92.3 a	2.2 c	9.0 c	23.5 b	1.4 c	*
Citronellol (3,7-dimethyloct-6-en-1-ol)	1205	100	5.3 d	22.1 c	26.0 c	127.8 a	130.1 a	134.2 a	16.7 c	23.4 c	22.1 c	48.7 b	65.4 b	103.5 a	19.2 c	**
Geraniol ((2E)-3,7-dimethylocta-2,6-dien-1-ol)	1258	75	nd	nd	19.3	nd	nd	nd	nd	nd	nd	33.8	nd	nd	nd	ns
Aldehydes [µg/L]																
Furfural (furan-2-carbaldehyde)	815	5000	13.2 c	14.9 c	14.7 c	17.1 c	86.7 a	22.2 c	15.3 c	109.3 a	46 b	11.3 c	12.1 c	18.5 c	11.8 c	*
Benzeneacetaldehyde (2-phenylacetaldehyde)	1015	5000	0.8	6.3	7.5	5.0	8.5	7.9	3.3	2.8	2.1	2.6	1.9	7.2	4.0	ns
Nonanal	1083	0.53	13.0 d	48.0 c	62.8 c	80.4 b	122.6 a	148.2 a	19.3 d	28.5 c	29.4 c	31.2 c	35.4 c	34.2 c	21.4 d	*
Acids [µg/L]																
Capric acid (Decanoic acid)	1360	2200	128.2	124.7	80.1	56.1	107.6	103.5	37.2	46.3	61.9	30.3	37.9	59.9	55.8	ns

^1^ LRI—linear retention index; the amount of components was determined; ^2^ Threshold in wine [29]. OAV—odour activity values (highlighted in green if OAV > 1); ^3^ Significance; ns—not statistically significant; *, **, *** indicate significance at a level of 0.05–0.01, 0.01–0.005 and <0.005, respectively, by the least significant difference; nd—not detected. Values with different letters (a–g) in the same raw indicate statistical differences.

**Table 5:** The odour intensity of key compounds shaping the aroma of apple wines that have been aged in the presence of wood.

Compounds	Ch1	Ch2	Ch3	A1	A2	A3	O1	O2	O3	M1	M2	M3	B	GC-O Descriptors ^1^
Isobutanol	0.5	1.0	1.0	1.0	1.5	1.0	0.5	1.0	1.0	0.5	1.0	1.0	1.0	Sweet, musty [FR, C]
Undecanol	0.5	0.5	0.5	0.0	0.0	0.0	0.0	0.0	0.0	0.0	0.0	0.0	0.0	Fruity, fatty [FR]
3-methyl 1-butanol	1.0	1.0	1.0	1.0	1.0	1.0	1.0	1.0	1.0	1.0	1.0	1.0	1.0	Sweet, pear [FR]
5-Hexen-1-ol	0.0	0.0	0.0	0.5	0.5	0.0	0.0	0.0	0.5	0.5	0.5	0.5	0.0	Grassy [H]
Phenylethanol	1.0	1.0	1.0	1.0	1.0	1.0	1.0	1.0	1.0	1.0	1.0	1.0	1.0	Rose [FL]
1-Hexanol	0.0	1.0	1.0	1.0	1.0	1.0	1.0	1.0	1.0	0.0	1.0	1.0	1.0	Winey, sweet, green [FR, H]
2-nonanol	0.0	0.0	1.3	0.0	0.0	0.0	0.0	0.0	0.0	1.0	0.0	0.0	0.0	fruity-green [FL, H]
Ethyl acetate	1.5	1.5	1.5	1.5	1.5	1.5	1.5	1.5	1.5	1.5	1.5	1.5	1.5	Fruity, pineapple [FR]
Methyl octanoate	0.0	0.0	0.0	1.0	1.0	1.0	1.0	1.0	1.0	0.0	0.0	0.0	0.0	winey, fruity, orange [FR]
Ethyl propanoate	1.0	1.0	1.0	1.0	1.3	1.0	1.3	1.0	1.3	1.0	1.0	1.3	1.3	Rummy, fruity [FR]
Methyl butanoate	1.0	1.0	1.0	1.0	1.0	1.0	1.0	1.0	1.0	1.0	1.0	1.0	1.0	Apple [FR]
Ethyl butanoate	1.0	1.0	1.0	1.3	1.3	1.3	0.0	0.0	0.0	0.0	0.0	0.0	1.0	Pineapple [FR]
2-methyl-ethyl butanoate	0.0	1.0	1.0	0.0	0.0	0.0	0.0	0.0	0.0	1.0	0.0	0.0	0.0	Fruity [FR]
1-Butanol, 2-methyl-acetate	0.0	1.0	1.0	0.0	0.0	0.0	0.0	0.0	0.0	1.0	0.0	0.0	0.0	Fruity, sweet, banana [FR]
Ethyl hexanoate	0.5	1.0	1.0	1.0	1.0	1.0	1.0	1.0	1.0	0.5	0.5	1.0	1.0	Apple peel [FR]
Diethyl butanoate	1.0	1.0	1.0	1.0	1.0	1.0	1.0	1.0	1.0	1.0	1.0	1.0	1.0	Winey-ethereal [H]
Ethyl decanoate	0.0	1.0	1.0	1.0	1.0	1.0	1.0	1.0	1.0	1.0	1.0	1.0	1.0	Banana, pear, apple [FR]
α-Pinene	1.0	0.0	1.0	0.0	0.0	0.0	0.0	1.3	0.0	0.0	0.0	0.0	0.0	Pine [H]
Linalool oxide	1.0	0.0	0.0	0.0	0.0	1.0	0.0	0.0	0.0	0.0	1.0	0.0	0.8	Earthy floral spice lavender [FL]
Citronellol	1.0	0.0	0.0	0.0	0.0	0.0	0.0	0.0	0.0	0.0	1.0	0.0	0.0	Rose [FL]
Geraniol	0.0	0.0	1.0	0.0	0.0	0.0	0.0	0.0	1.0	0.0	0.0	0.0	0.0	Sweet, rose [FL]
trans-Farnesol	0.0	0.0	0.0	0.0	0.0	0.0	0.0	0.0	1.0	0.0	0.0	0.0	0.0	Woody, floral [FL, R]
Furfural	1.0	1.0	1.0	1.0	1.0	1.0	1.0	1.0	1.0	1.0	1.0	1.0	1.0	Almond [R]
Benzene acetaldehyde	1.0	1.0	1.0	1.0	1.0	1.0	1.0	1.0	1.0	1.0	1.0	1.0	1.0	Grassy [H]
Nonanal	1.0	0.0	0.0	0.0	0.0	0.0	0.0	0.0	0.0	0.0	0.0	0.0	0.0	Rose, citrus [FL, FR]

The lowest odour intensity in these columns is marked as dark red, while the highest intensity is marked as the darkest green. SD < 5%; Ch—cherry wood chips, A—apple wood chips, O—oak wood chips, M—maple wood chips; 1—lightly toasted, 2—medium-toasted, 3—heavily toasted. ^1^ The aroma descriptor was perceived at the sniffing port of the GC-O. The aroma group of detected aroma descriptors was signed by letters in brackets as follows: roasted [R], fruity [FR], floral [FL], herbaceous [H] and chemical [C].

## Data Availability

Data are contained within the article.
